# Comprehensive analysis of two hotspot codons in the *TUBB4B* gene and associated phenotypes

**DOI:** 10.1038/s41598-024-61019-0

**Published:** 2024-05-08

**Authors:** Jan-Philipp Bodenbender, Valerio Marino, Julia Philipp, Anke Tropitzsch, Christoph Kernstock, Katarina Stingl, Melanie Kempf, Tobias B. Haack, Theresia Zuleger, Pascale Mazzola, Susanne Kohl, Nicole Weisschuh, Daniele Dell’Orco, Laura Kühlewein

**Affiliations:** 1 University Eye Hospital, Centre for Ophthalmology, University of Tübingen, Tübingen, Germany; 2 Department of Neurosciences, Biomedicine and Movement Sciences, Section of Biological Chemistry, University of Verona, Verona, Italy; 3 Department of Otolaryngology-Head & Neck Surgery, Hearing Research Center, University of Tübingen Medical Center, Tübingen, Germany; 4 Centre for Rare Diseases, University of Tübingen, Tübingen, Germany; 5 Institute of Medical Genetics and Applied Genomics, University of Tübingen, Tübingen, Germany; 6 Molecular Genetics Laboratory, Institute for Ophthalmic Research, Centre for Ophthalmology, University of Tübingen, Tübingen, Germany; 7 Institute for Ophthalmic Research, Centre for Ophthalmology, University of Tübingen, Tübingen, Germany

**Keywords:** *TUBB4B*, Hereditary retinal dystrophy, Leber congenital amaurosis, Retinitis pigmentosa, Hearing loss, Structural analysis, Gene expression, Genetics research

## Abstract

Our purpose was to elucidate the genotype and ophthalmological and audiological phenotype in *TUBB4B*-associated inherited retinal dystrophy (IRD) and sensorineural hearing loss (SNHL), and to model the effects of all possible amino acid substitutions at the hotspot codons Arg390 and Arg391. Six patients from five families with heterozygous missense variants in *TUBB4B* were included in this observational study. Ophthalmological testing included best-corrected visual acuity, fundus examination, optical coherence tomography, fundus autofluorescence imaging, and full-field electroretinography (ERG). Audiological examination included pure-tone and speech audiometry in adult patients and auditory brainstem response testing in a child. Genetic testing was performed by disease gene panel analysis based on genome sequencing. The molecular consequences of the substitutions of residues 390 and 391 on TUBB4B and its interaction with α-tubulin were predicted in silico on its three-dimensional structure obtained by homology modelling. Two independent patients had amino acid exchanges at position 391 (p.(Arg391His) or p.(Arg391Cys)) of the TUBB4B protein. Both had a distinct IRD phenotype with peripheral round yellowish lesions with pigmented spots and mild or moderate SNHL, respectively. Yet the phenotype was milder with a sectorial pattern of bone spicules in one patient, likely due to a genetically confirmed mosaicism for p.(Arg391His). Three patients were heterozygous for an amino acid exchange at position 390 (p.(Arg390Gln) or p.(Arg390Trp)) and presented with another distinct retinal phenotype with well demarcated pericentral retinitis pigmentosa. All showed SNHL ranging from mild to severe. One additional patient showed a variant distinct from codon 390 or 391 (p.(Tyr310His)), and presented with congenital profound hearing loss and reduced responses in ERG. Variants at codon positions 390 and 391 were predicted to decrease the structural stability of TUBB4B and its complex with α-tubulin, as well as the complex affinity. In conclusion, the twofold larger reduction in heterodimer affinity exhibited by Arg391 substitutions suggested an association with the more severe retinal phenotype, compared to the substitution at Arg390.

## Introduction

The most common cause of deafblindness with a prevalence of 1:30,000 is Usher syndrome (USH)^1^. In this syndrome, sensorineural hearing loss (SNHL) and retinitis pigmentosa (RP) occur in a syndromic conjunction^1^. Hearing loss is congenital or manifests in childhood or early teens, while visual impairment is diagnosed later. Three types of USH are distinguished: type 1 accounts for ~ 40% of cases and includes congenital profound hearing loss and RP beginning in childhood. USH type 2 is the most common form at ~ 60% and includes congenital moderate to severe and slowly progressive hearing loss and RP with an onset at age 20. USH type 3 accounts for < 3% of cases and is characterized by rapidly progressive postlingual hearing loss and RP with an onset in the second decade^1,2^. Inheritance is autosomal recessive. USH type 1 is associated with biallelic disease-causing variants in the genes *MYO7A*, *USH1C*, *CDH23*, *PCDH15*, *USH1G*, and *CIB2*, type 2 in *USH2A*, *ADGRV1*, and *WHRN*, and type 3 in *CLRN1* and *HARS*, respectively^1^. However, the link between the gene affected and the type is not rigid^1^.

A rare cause of deaf-blindness is Leber congenital amaurosis with early-onset deafness (LCAEOD; OMIM #617879), which only recently has been described by Luscan and coworkers^3–5^. Patients present with early-onset centrally-progressing retinal degeneration and hearing deficiencies due to cochlear cell loss. Symptoms encompass nyctalopia, visual field constriction and severe SNHL^3^. To date, LCAEOD has only been associated with two heterozygous (monoallelic) missense variants in the *TUBB4B* gene (tubulin beta 4B class IVb), namely c.1171C>T;p.(Arg391Cys) and c.1172G>A;p.(Arg391His)^3–5^. In familial cases, the inheritance pattern was shown to be autosomal dominant, but most cases were de novo^3–5^. Symptoms occur within the first decade of life^3,5^.

**Figure 1:**
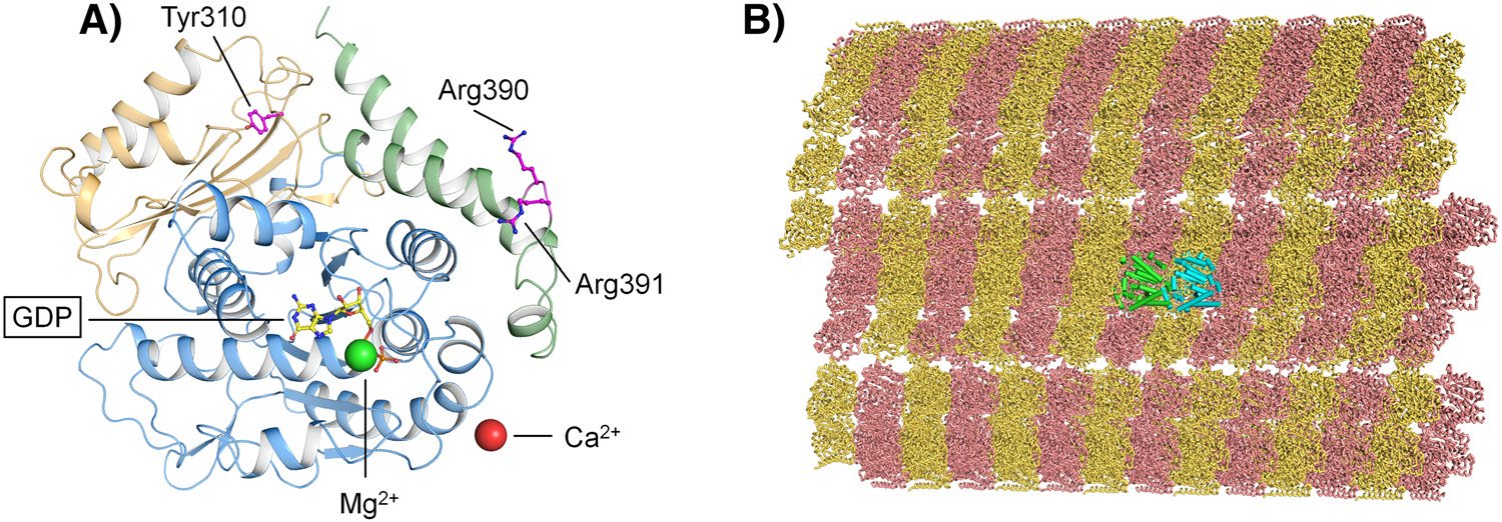
(**A**) Three-dimensional structure of GDP-bound TUBB4B. Protein structure is displayed as cartoons with the N-terminal tubulin/FtsZ GTPase domain in blue, the tubulin/FtsZ C-terminal domain in orange and the helix hairpin bin motif in green. GDP is framed and represented as sticks with C atoms in yellow, P atoms in orange, N atoms in blue and O atoms in red. Mg^2+^ and Ca^2+^ ions are displayed as green and red spheres, respectively. The sidechains of residues whose disease-associated variants were found in this patient cohort are labelled and represented as sticks with C atoms in purple, O atoms in red and N atoms in blue. (**B**) Structural organization of a microtubule (PDB entry: 7UNG) with α-tubulin molecules represented as yellow ribbons and β-tubulin molecules as pink ribbons. The repeating unit constituted by the αβ-tubulin het-erodimer is shown as cylindrical cartoons with α-tubulin in cyan and β-tubulin in green.

The *TUBB4B* gene (OMIM *602660) is located on chromosome 9q43.3 and encodes the 445 amino acids-comprising beta-tubulin 4B chain protein (TUBB4B), which has been shown to be highly expressed in the mouse retina^3^. TUBB4B is an αβ-class protein belonging to the tubulin/FtsZ/CetZ-like superfamily and it is constituted by three structural domains (Fig. 1A), the N-terminal tubulin/FtsZ GTPase domain (residues 1–260), the tubulin/FtsZ C-terminal domain (residues 261–373),and the helix hairpin bin motif (residues 374–427), and a negatively charged disordered C-terminus (residues 428–445). Heterodimers of ß-tubulin and α-tubulin form microtubules (Fig. 1B), which partake in mitosis, intracellular transport, neuron morphology, axon guidance, ciliary and flagellar motility^6^. The two known disease-causing missense variants in TUBB4B locate to amino acid position 391. In the orthologous ß-tubulin protein encoded by *TUBB2B*, residue Arg391 and the adjacent residues Arg390 and Lys392 were shown to form a binding pocket that interacts with α-tubulin in the longitudinally adjacent tubulin αß-heterodimer^7^. It has been suggested that the substitution of arginine at position 391 with histidine or cysteine destabilizes hydrophobic interactions in the adjacent αß-heterodimer^8^. This predicted destabilization is consistent with a diminished microtubule growth rate that was observed in patient-derived skin fibroblast of patients harboring the c.1171C>T;p.(Arg391Cys) and c.1172G>A;p.(Arg391His) variants in *TUBB4B*^3^. Only recently another disease-related variant in the *TUBB4B* gene was described (c.32A>G;(p.(Gln11Arg)). Phenotypically, this was associated with early-onset hearing loss and hyperopia, but without retinal changes. Furthermore, the patient exhibited hypophosphatemic rickets (HR), renal tubular Fanconi syndrome (FS), and nephrocalcinosis^9^.

In this study, we aimed to describe a cohort of patients with inherited retinal dystrophy (IRD) and SNHL harboring known and novel missense variants in *TUBB4B*. In addition, we used a computational structural approach to examine the effects of all theoretically possible amino acid substitutions at codons 390 and 391.

## Results

Five patients with *TUBB4B*-codon Arg390/Arg391 missense variants associated with IRD and SNHL were included in the study (Table 1). In addition, a patient with a novel heterozygous missense variant of uncertain significance c.928T>C;p.(Tyr310His) and early-onset deafness (EOD) was also included. The mean age of the patients was 32.8 years (range, 1–69 years). Two patients were female. Patient 1 and Patient 2 are son and mother; the other patients are not related.

**Table 1:** Patient demographics, genotype and phenotypic summary.

ID	Age*	*Sex*	*TUBB4B* genotype	HGMD accession no	Retinal disease*	Hearing impairment (PTA4)
1	39	m	c.1169G>A p.(Arg390Gln) Heterozygous	–	Diagnosis at 25 years; BCVA: R/L 20/20 (R − 0.75/− 0.75 × 2°, L − 0.50/− 0.75 × 1°); VF: ring scotoma 5–25°; ERG: reduced scotopic and photopic responses; Fundus: well demarcated pericentral RP	mild HL R 20.75 dB HL L 20.50 dB HL
2	69	f	c.1169G>A p.(Arg390Gln) Heterozygous	–	Diagnosis at 39 years; BCVA: R/L HM (R + 0.75/− 0.75 × 95°, L − 0.50/− 2.25 × 78°); VF: irregularly narrowed to 5–25°; ERG: reduced scotopic and photopic responses; Fundus: well demarcated pericentral RP	moderate HL R 44.25 dB HL L 43.75 dB HL
3	36	m	c.1168C>T p.(Arg390Trp) heterozygous	–	Diagnosis at 36 years; BCVA: R 20/20, L 20/160 (R + 1.00/− 0.75 × 13°; L + 3.50/− 2.25 × 2°), L amblyopia; VF: ring scotoma 10–30°; ERG: reduced scotopic and photopic responses; Fundus: ring-shaped atrophy around the posterior pole adjacent to the arcades	severe HL R 66.25 dB HL L 65.00 dB HL
4	28	m	c.1172 = /G>A p.(Arg391 = /His)	CM1716631	Diagnosis at 17 years; BCVA: R 20/40 L 20/60 (R + 3.25/− 2.50 × 15°, L + 3.25/− 3.00 × 174°); VF: R superior visual field defect L outer borders essentially normal; ERG: reduced scotopic and no photopic responses; Fundus: map-like atrophic areas throughout the retina with bone spicules in the more peripheral lesions	mild HL R 25.00 dB HL L 28.75 dB HL
5	24	m	c.1171C>T p.(Arg391Cys) Heterozygous	CM1716632	Diagnosis at 4 years; BCVA: R LP L NLP* (R + 8.25/− 1.25 × 21°, L + 8.50/− 1.50 × 171°)**; ERG: no photopic responses; Fundus: pigment deposits in the macula and peripherally inside small round atrophic lesions	moderate HL R 41.25 dB HL L 45.00 dB HL
6	1	f	c.928T>C p.(Tyr310His) Heterozygous	–	Diagnosis at 1 year; BCVA: R/L 0.63 decimal in 0.84 m distance (R + 3.50/ + 2.50 × 90°; L + 3.50/ + 2.50 × 80°); ERG: reduced scotopic and photopic responses; fundus: light-colored retina	congenital profound bilateral HL: no potentials in ABR in air and bone conduction

*At the most recent presentation; **values from previous exam at age 4 years.; m: male; f: female; R: right eye; L: left eye; BCVA: best corrected visual acuity; VF: visual field; ERG: electroretinogram; HL: hearing loss; PTA4: Pure Tone Average (PTA) threshold for frequencies of 0.5, 1, 2, and 4 kHz; ABR: auditory brainstem response.

### Clinical and genetic findings

#### Patient 1

**Figure 2:**
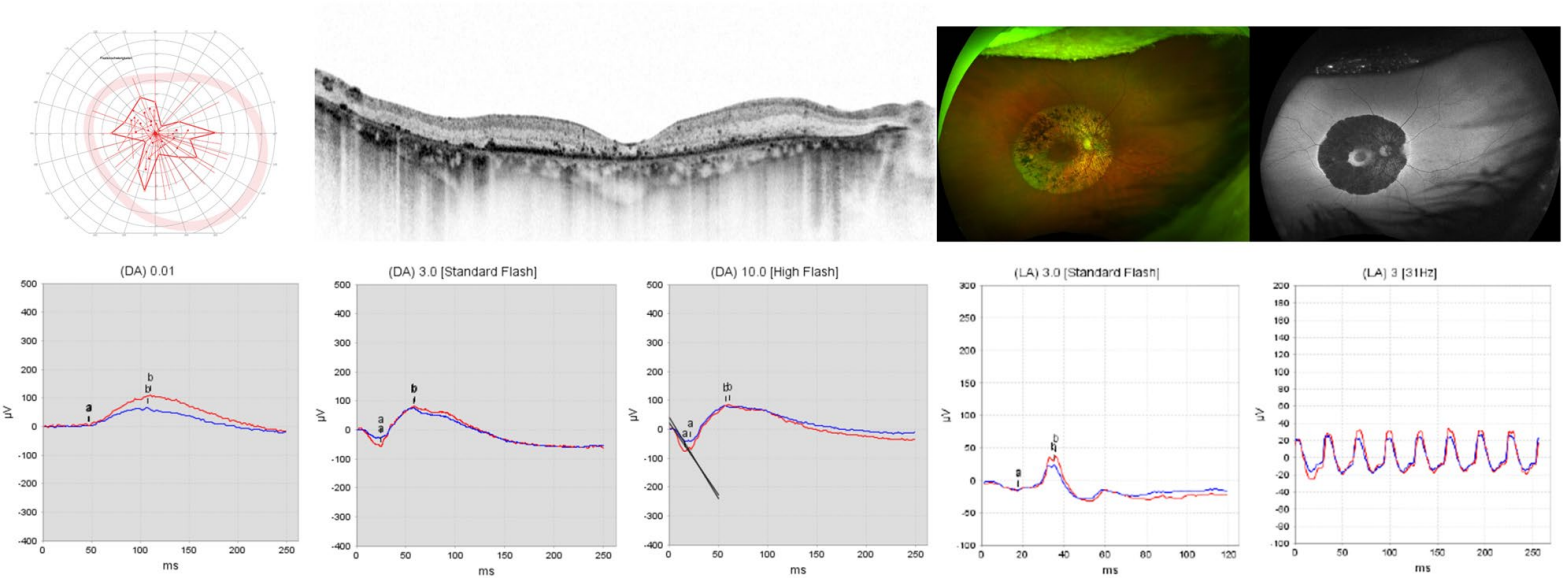
Patient 1: 39 y.o. male, best-corrected visual acuity 20/20 Snellen equivalent in both eyes. Genotype TUBB4B c.1169G>A;p.(Arg390Gln), heterozygous. From upper left to lower right: kinetic visual field, optical coherence tomography, fundus and fundus autofluorescence imaging of the right eye and electroretinogram under scotopic and photopic testing conditions of both eyes.

Patient 1 presented to us at the age of 39 years. He reported nyctalopia as a first symptom at the age of 25 years, glare sensitivity and visual field defects. Medical history was unremarkable. Best corrected visual acuity (BCVA) was 20/20 in both eyes. The anterior segment and intraocular pressure (IOP) were within normal limits. Posterior segment examination revealed narrow vessels and atrophy and few bone spicules along the arcades (Fig. 2). Kinetic perimetry using target III4e revealed a ring-scotoma between 5° and 25°. Optical coherence tomography (OCT) showed atrophy of the outer retina except for a small foveal area with preserved ellipsoid zone (EZ). Fundus autofluorescence (FAF) imaging showed a parafoveal hyperautofluorescent ring and, more remarkably, patchy decreased autofluorescence along the arcades surrounded by an hyperautofluorescent ring. Electroretinography (ERG) revealed reduced amplitudes under scotopic and under photopic testing conditions. Ophthalmological findings were highly symmetrical.

The patient did not report any hearing difficulties. Pure tone audiometry (PTA4) revealed bilateral mild hearing loss (20.75 dB HL right ear; 20.50 dB HL left ear). Speech audiometry (Freiburg Test) showed a speech recognition threshold (SRT) of 12 dB for both the right and the left ear; word recognition score WRS65 was 100% for the right and the left ear. Transient otoacoustic emissions (TEOAEs) and distortion-product OAEs (DPOAEs) were reproducible. Brainstem audiometry using a Chirp-stimulus confirmed bilateral detection threshold for Jewett wave V at 20 dB.

The patient was shown to be heterozygous for a novel missense *TUBB4B* variant, c.1169G>A;p.(Arg390Gln). No other pathogenic or likely pathogenic or other candidate variants of uncertain significance were prioritized in the diagnostic genetic testing.

#### Patient 2

**Figure 3:**
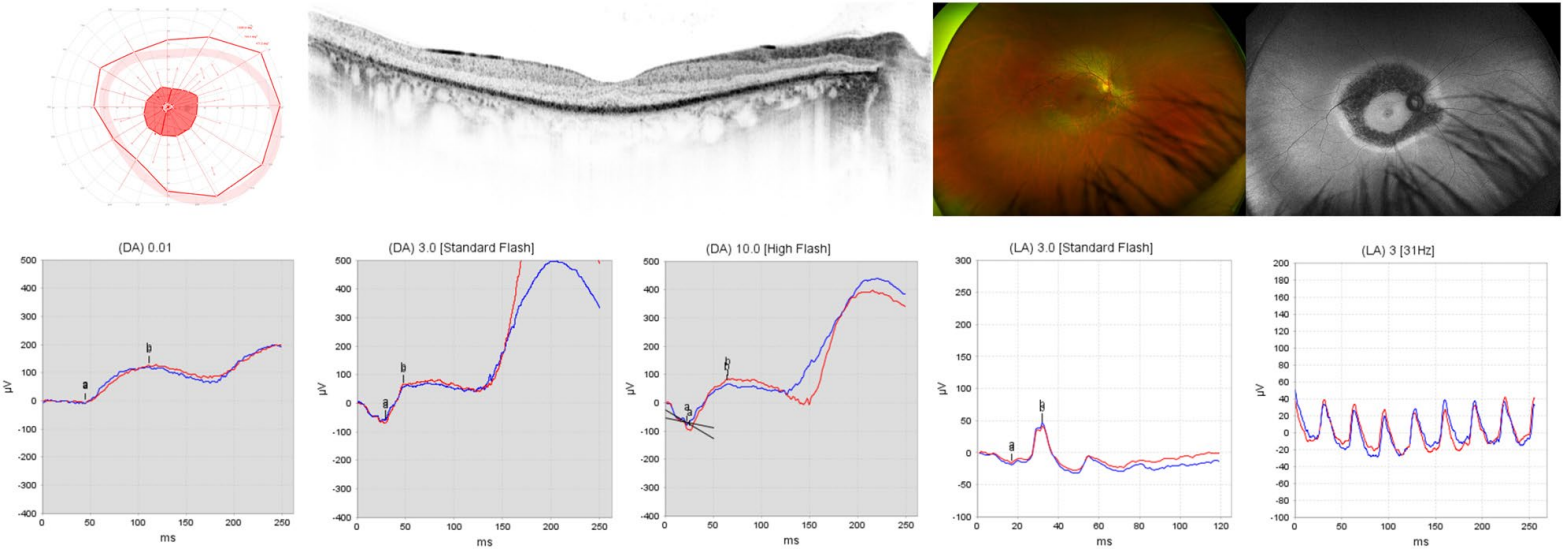
Patient 2: 69 y.o. female, best-corrected visual acuity was hand movements in both eyes. Genotype TUBB4B c.1169G>A;p.(Arg390Gln), heterozygous. From upper left to lower right: kinetic visual field, optical coherence tomography, fundus and fundus autofluorescence imaging of the right eye and electroretinogram under scotopic and photopic testing conditions of both eyes.

Patient 2, the mother of Patient 1, presented to us at the age of 69 years. Nyctalopia was the first symptom with an onset in her mid-30s. Central visual acuity was affected at the age of 50. She was first diagnosed at the age of 39. Medical history was remarkable for hypothyroidism. BCVA was hand movements in both eyes. The anterior segment and IOP were unremarkable, apart from an incipient cataract. Posterior segment examination revealed narrow vessels and ring-shaped atrophy with multiple bone spicules extending from the parafovea to the arcades and in the left eye additionally asteroid hyalosis (Fig. 3). Kinetic perimetry using target III4e revealed an irregularly narrowed visual field probably due to the low visual acuity and fixation difficulties. OCT imaging showed atrophy of the outer retina with EZ loss also centrally. FAF imaging showed decreased autofluorescence extending from the parafovea to the arcades surrounded by an hyperautofluorescent ring. ERG testing revealed reduced amplitudes under scotopic and photopic testing conditions. Ophthalmological findings were highly symmetrical.

The patient’s otologic history was remarkable for hearing loss. PTA4 revealed bilateral moderate hearing loss (44.25 dB HL right ear; 43.75 dB HL left ear). In speech audiometry (Freiburg Test), the SRT was 38 dB for the right ear and 35 dB for the left ear; WRS65 was 70% for the right ear and 75% for the left ear. TEOAEs and DPOAEs were absent. Brainstem audiometry using a Chirp-stimulus confirmed bilateral detection threshold for Jewett wave V at 40 dB.

Segregation analysis by PCR amplification of *TUBB4B* exon 4 and Sanger-sequencing proved her to also be heterozygous for the novel missense *TUBB4B* variant, c.1169G>A;p.(Arg390Gln).

#### Patient 3

**Figure 4:**
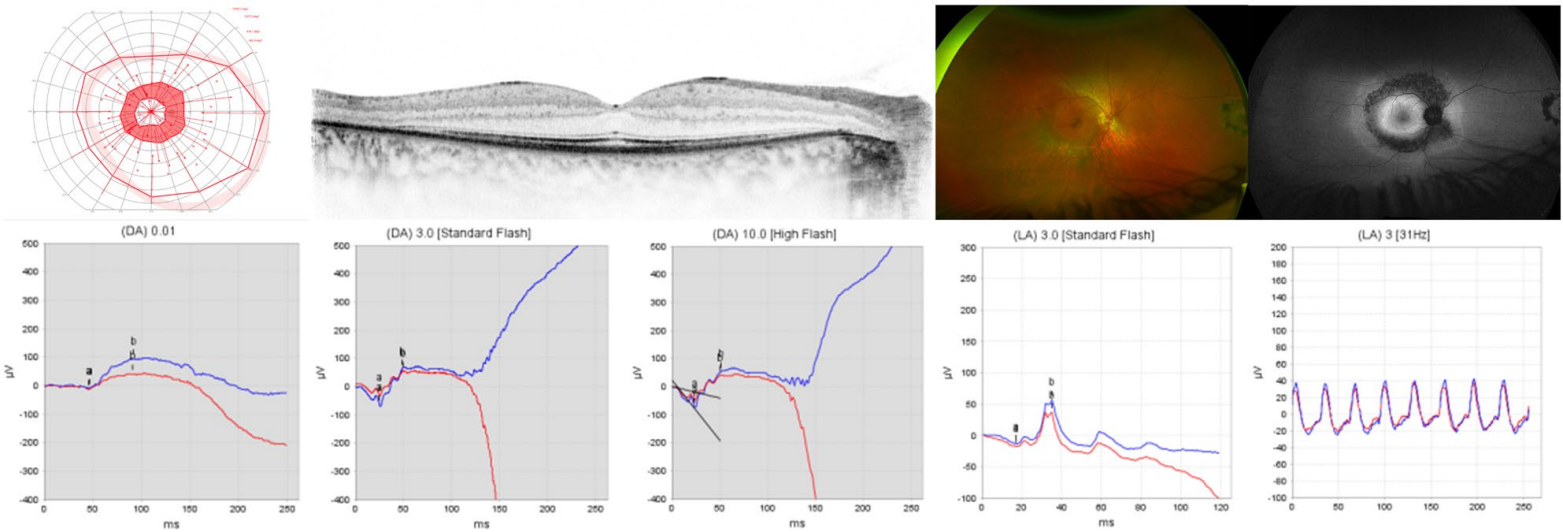
Patient 3: 36 y.o. male, best-corrected visual acuity 20/20 Snellen equivalent in the right eye and 20/160 in the left eye. TUBB4B genotype c.1168C>T;p.(Arg390Trp), heterozygous. From upper left to lower right: Kinetic visual field, optical coherence tomography, fundus and fundus autofluorescence imaging of the right eye and electroretinogram under scotopic and photopic testing conditions of both eyes.

Patient 3 presented to us at the age of 36 years. He reported glare sensitivity, but no other symptoms. His left eye was amblyopic, and the right eye had been laser-treated due to a retinal hole in the nasal periphery. Medical history was unremarkable. BCVA was 20/20 in the right eye and 20/160 in the left eye. The anterior segment and IOP were within normal limits. Posterior segment examination revealed somewhat narrowed vessels and ring-shaped atrophy around the posterior pole adjacent to the arcades (Fig. 4). Kinetic perimetry using target III4e revealed a ring-scotoma between 10 and 30°. OCT imaging showed thinning of the outer retinal layers beyond 20°. FAF imaging showed a hyperautofluorescent ring around the fovea, decreased autofluorescence around the posterior pole adjacent to the arcades, joined by another ring of hyperautofluorescence outside of which the FAF signal was normal. ERG testing revealed reduced amplitudes under scotopic and (to a lesser extent) under photopic testing conditions. Ophthalmological findings except for BCVA were highly symmetrical.

Otologic history was remarkable for hearing loss. PTA4 revealed bilateral severe hearing loss (66.25 dB HL right ear; 65.00 dB HL left ear). Speech audiometry (Freiburg Test) showed an SRT of 56 dB for both the right and the left ear; WRS65 was 45% for the right ear and 25% for the left ear.

The patient was shown to be heterozygous for a novel *TUBB4B* missense variant, c.1168C>T;p.(Arg390Trp). No other pathogenic or likely pathogenic or other candidate variants of uncertain significance were prioritized in the diagnostic genetic testing.

#### Patient 4

**Figure 5:**
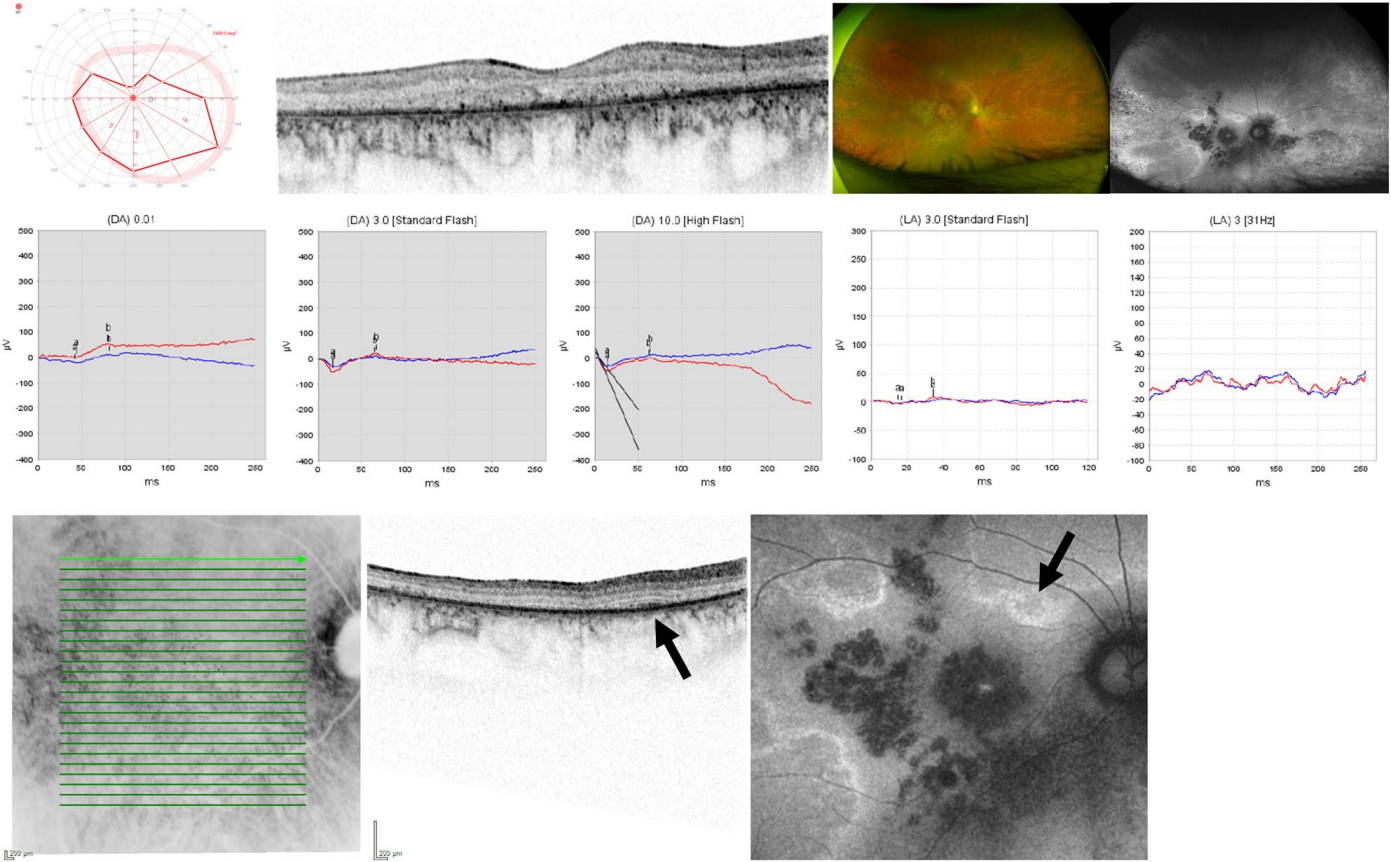
Patient 4: 28 y.o. male, best-corrected visual acuity 20/40 Snellen equivalent in the right eye and 20/60 in the left eye. TUBB4B genotype c.1172 = /G>A;p.(Arg391 = /His). From upper left to mid right: Kinetic visual field, optical coherence tomography (OCT), fundus and fundus autofluorescence (AF) imaging of the right eye and electroretinogram under scotopic and photopic testing conditions of both eyes. Lower left: infrared reflectance and OCT image of the upper macular region of the right eye with corresponding AF image (lower right). Note the arrow indicating a less affected area indicating mosaicism at the level of the retinal tissue.

Patient 4 presented to us most recently at the age of 28 years. He reported nyctalopia and glare sensitivity since the age of 17, when the patient presented to us for the first time, accompanied by progressive visual field defects. Medical history was unremarkable. BCVA was 20/40 in the right eye and 20/60 in the left eye. The anterior segment was unremarkable. Posterior segment examination revealed narrow vessels and map-like atrophic areas throughout the retina with bone spicules in the more peripheral lesions (Fig. 5). Kinetic perimetry using target III4e revealed a superior visual field defect in the right eye, while outer borders were essentially normal in the left eye. OCT imaging showed atrophy of the outer retina with EZ loss also centrally. FAF imaging showed again map-like lesions with hyperfluorescent borders, round spots of decreased autofluorescence centrally, and patches of decreased autofluorescence with a spicule-like pattern inside more peripherally. ERG testing revealed markedly reduced responses under scotopic testing conditions and essentially no responses under photopic testing conditions. Comparison of his clinical data showed progression in that the patient most recently reported night blindness. This was supported by ERG testing, which then had shown higher amplitudes under scotopic testing conditions and a small but detectable 30 Hz flicker response under photopic testing conditions. Accordingly, OCT had shown better organized outer retinal layers at first presentation.

Otologic history was remarkable for stable hearing loss since the age of 17 years. PTA4 revealed a bilateral mild hearing loss (25.00 dB HL right ear; 28.75 dB HL left ear) with a mid-frequency hearing loss configuration.

The patient was shown to be heterozygous for the known *TUBB4B* variant c.1172G>A;p.(Arg391His). Of note, the genetic testing by genome sequencing revealed a reduced number of reads for the variant, indicating a mosaicism of 24% for the variant in genomic DNA isolated from leukocytes (Supplementary Fig. S1). Mosaicism was confirmed from an independent blood sample (Supplementary Fig. S1) as well as a mucosal swap (data not shown), but the fraction was not determined. The variant has likely arisen as a de novo mutational event as there was no family history of IRD or SNHL, and segregation analysis did not display the variant in neither of the parents. In addition, the patient was shown to be heterozygous for a pathogenic variant in the *MYO15A* gene (c.1185dup;p.(Glu396ArgfsTer36)). Biallelic pathogenic variants in *MYO15A* are associated with autosomal recessive SNHL. Yet, no additional variant in *MYO15A* was identified, suggesting only a carrier status for this variant in the patient.

#### Patient 5

**Figure 6:**
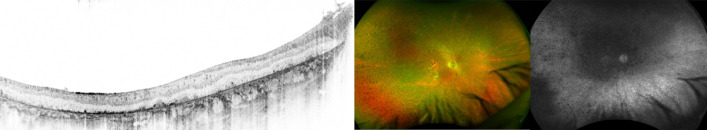
Patient 5: 24 y.o. male, best-corrected visual acuity was light perception in the right eye and no light perception in the left eye. TUBB4B genotype c.1171C>T;p.(Arg391Cys), heterozygous. From left to right: Optical coherence tomography, fundus and fundus autofluorescence imaging of the right eye. Note the ubiquitous changes in the fundus of the patient.

Patient 5 presented to us most recently at the age of 24 years. He reported nyctalopia and reduced color and contrast vision as the first symptom at the age of 4 years, when the patient presented to us for the first time. Medical history was unremarkable. BCVA was light perception in the right eye and no light perception in the left eye. The anterior segment was within normal limits. Posterior segment examination revealed a waxy optic disc, narrow vessels, and pigment deposits in the macula and peripherally inside small round atrophic lesions (Fig. 6). Kinetic perimetry was not possible due to the low visual acuity. OCT imaging showed atrophy of the outer retina with EZ loss also centrally. FAF imaging showed decreased autofluorescence at the posterior pole extending to the arcades and round hyper- and hypoautofluorescent lesions in the midperiphery and periphery, temporally pronounced. Ophthalmological findings were highly symmetrical. Comparison of his clinical data showed progression in that the patient then had a BCVA of 20/50 in both eyes (R + 8.25/−1.25 × 21°; L + 8.50/− 1.50 × 171°). ERG testing revealed no responses under photopic testing conditions already at the age of 4 years.

Otologic history was remarkable for hearing loss. PTA4 revealed bilateral moderate hearing loss (41.25 dB HL right ear; 45.00 dB HL left ear) in a high-frequency downsloping configuration. Speech audiometry (Göttinger test) showed a WRS65 of 70% for the right ear and 80% for the left ear. Hearing aid fitting was recommended.

The patient was shown to be heterozygous for the known *TUBB4B* missense variant c.1171C>T;p.(Arg391Cys). In addition, the patient was shown to be heterozygous for a variant of uncertain significance in *TOPORS* c.2347G>A;p.(Gly783Arg). Heterozygous pathogenic variants in *TOPORS* cause autosomal dominant RP. Yet there was no family history of RP in this patient, and the variant has never been associated with any IRD and was additionally observed once in a population database in an African population (i.e. gnomAD). Prediction programs suggest a benign effect, although it affects an evolutionary conserved amino acid residue.

#### Patient 6

**Figure 7:**
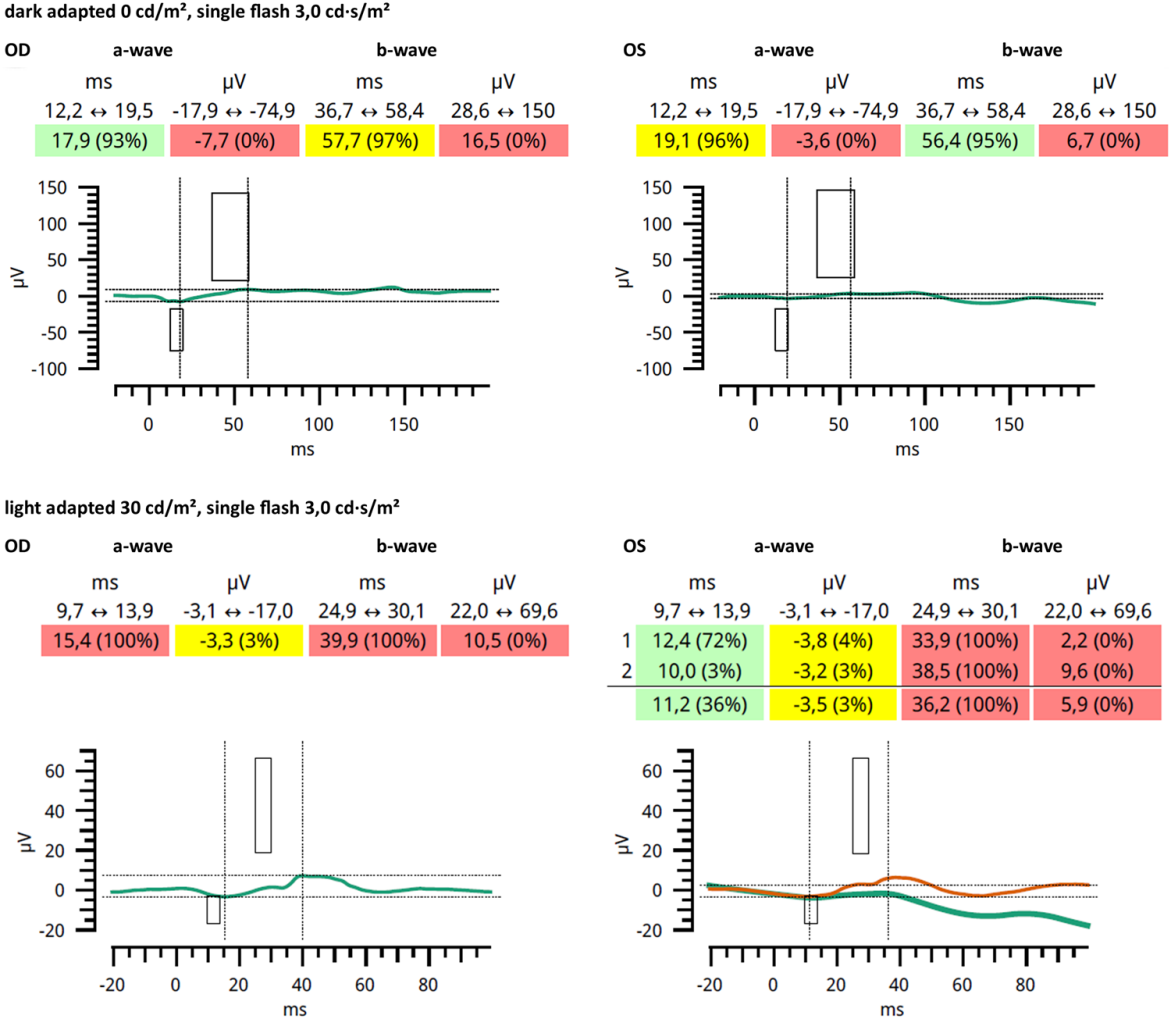
Patient 6: Electroretinogram under general anesthesia using the RETeval device and skin electrodes for both eyes. Upper panel: Responses under scotopic testing conditions for the right eye (OD) and left eye (OS); background 0.0 cd/m^2^, single flash 3.0 cd·s/m^2^. Lower panel: Responses under photopic testing conditions; background 30 cd/m^2^, single flash 3.0 cd·s/m^2^. Green and orange lines indicate the responses, the boxes indicate normative values.

Patient 6 presented to the Department of Otolaryngology-Head & Neck Surgery at the age of 3 months. Her parents had not noticed any signs of visual impairment. Medical history was unremarkable. Binocular visual acuity testing with Teller Acuity Cards revealed an age appropriate BCVA of 0.63 decimal in 0.84 m distance without a side preference. The anterior segment and IOP were within normal limits. Posterior segment examination revealed a yellowish/hyperemic optic disc with slightly blurred edges and a light-colored retina. Binocular LED confrontation perimetry in 1 m distance revealed no sign of a visual field defect. OCT and FAF imaging were not possible due to the (young) age of the patient. ERG testing under general anesthesia using the RETeval device and skin electrodes revealed reduced amplitudes under scotopic and photopic testing conditions (Fig. 7).

The patient suffered from congenital bilateral profound hearing loss. No potentials in auditory brainstem response (ABR) in air and bone conduction were detectable. Hearing aids in both ears and bilateral cochlear implantation were indicated.

Genetic diagnostic testing revealed a novel heterozygous *TUBB4B* missense variant c.928T>C;p.(Tyr310His). No other pathogenic or likely pathogenic or other candidate variants of uncertain significance for SNHL were prioritized in the diagnostic genetic testing. Segregation analysis did not detect the variant in the parents. Therefore, a de novo mutation is assumed. This rare variant has neither been described in association with any inherited disease nor has it been observed in any population databases (i.e. gnomAD). It affects a highly conserved amino acid residue at the beginning of β8 strand of TUBB4B, and is predicted to be disease-related by various prediction software and was therefore prioritized in the genetic work-up.

### Molecular modeling of structural variants

**Figure 8:**
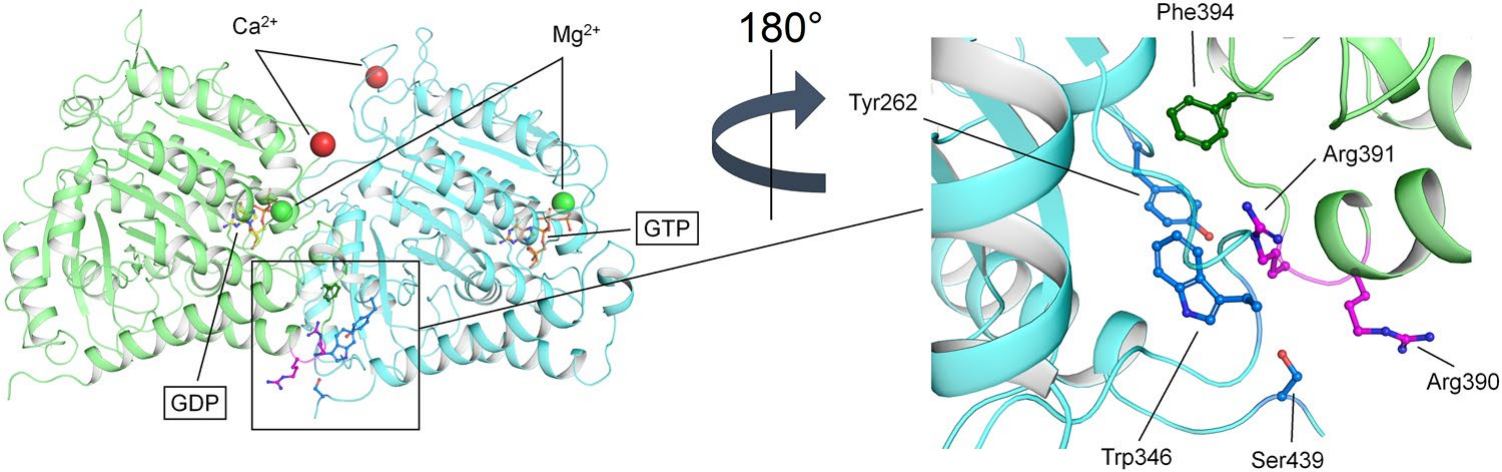
Interface of the αβ-tubulin heterodimer. The three-dimensional structure of the αβ-tubulin heterodimer is represented as cartoon with α-tubulin in cyan and β-tubulin in green. GDP and GTP are labelled, framed, and represented as sticks with C atoms in yellow and light orange, respectively, P atoms in orange, N atoms in blue and O atoms in red. Ca^2+^ and Mg^2+^ ions are shown as red and green spheres, respectively. The sidechains of residues whose disease-associated variants were found in this patient cohort are labelled and represented as sticks with C atoms in purple, O atoms in red and N atoms in blue. The sidechains of the residues belonging to the dimer interface and within 5 Å from either Arg390 or Arg391 are labelled and represented as sticks with C atoms in dark blue and dark green for residues of α- and β-tubulin, respectively, N atoms in blue and O atoms in red. Inset represents a detailed view of the dimer interface after a 180° rotation on the Z axis.

*TUBB4B* encodes for a 49.8 KDa (445 residues) protein which is constituted by 12 α-helices (α1 to α12) and 10 β-strands (β1 to β10), typical of an αβ-class protein. The N-terminal tubulin/FtsZ GTPase domain (residues 1–260, Fig. 1A) is arranged in a 3-layer (aba) sandwich architecture organized in a Rossmann fold, where strands β1 to β6 constitute a β-sheet sandwiched between parallel helices α1-α2 and α4-α5. The tubulin/FtsZ C-terminal domain (residues 261–373), on the other hand, is spatially organized in a 2-layer sandwich architecture where strands β7 to β10 constitute a β-sheet sandwiched against helices α9-α10, and it hosts one of the residues found mutated in our patient cohort, namely Tyr310, which is located at the beginning of β8 strand (Fig. 1A). Finally, the helix hairpin bin motif (residues 374–427) is constituted by helices α11 and α12 connected by a loop in which residues Arg390 and Arg391 are located (Fig. 1A). These disease-associated missense variants were found in our patient cohort. Interestingly, residues Arg390 and Arg391 reside on the interface between the α and β subunits, with potential implications in microtubule polymerization (Fig. 8).

We performed an in silico investigation to evaluate the molecular consequences of the five TUBB4B amino acid substitution variants found in our patient cohort, namely Tyr310His, Arg390Gln, Arg390Trp, Arg391Cys and Arg391His, in terms of differences in apparent stability of the isolated protein (∆∆G_f_^app^) and in apparent affinity (∆∆G_b_^app^) for the α subunit (Fig. 8). Moreover, the same analysis was performed on all possible missense variants due to point mutations in the codons encoding for residues Arg390 (to Gly, Leu, and Phe) and Arg391 (to Gly, Leu, Phe, and Ser), to discriminate in which position the amino acid substitution is more detrimental to microtubule stability and elongation, thus allowing for investigating potential genotype/phenotype correlations.

All observed five variants exhibited a destabilization of the folding of the isolated protein (Table 2), with the Tyr310His variant showing a milder effect (∆∆G_f_^app^ = 3.13 kcal/mol) compared to the variants Arg390Gln or Arg390Trp (∆∆G_f_^app^ = 8.27 and 9.59 kcal/mol, respectively) and Arg391Cys or Arg391His (∆∆G_f_^app^ = 10.81 and 9.26 kcal/mol, respectively).

**Table 2:** Effects of TUBB4B missense variants on the apparent relative stability of the isolated protein and in complex with α-tubulin (∆∆G_f_^app^), and their affinity (∆∆G_b_^app^).

TUBB4B variant	∆∆G_f_^app^ (kcal/mol)		∆∆G_b_^app^ (kcal/mol)
	Isolated monomer	αβ heterodimer	αβ heterodimer
Tyr310His	3.13	3	0.01
Arg390Gln	8.27	17.52	8.66
*Arg390Gly*	*15.8*	*23.6*	*12.13*
*Arg390Leu*	*5.78*	*13.17*	*10.3*
*Arg390Pro*	*50.93*	*58.24*	*11.78*
Arg390Trp	9.59	19.08	8.36
**Arg390**	**15.58 ± 17.83**	**26.32 ± 18.23**	**10.25 ± 1.73**
Arg391Cys	10.81	16.27	21.8
*Arg391Gly*	*15.07*	*24.23*	*24.39*
Arg391His	9.26	21.25	20.95
*Arg391Leu*	*4.27*	*16*	*15.99*
*Arg391Pro*	*32.16*	*36.72*	*20.25*
*Arg391Ser*	*13.41*	*20.7*	*22.86*
**Arg391**	**14.16 ± 9.57**	**22.53 ± 7.63**	**21.04 ± 2.87**

Values for Arg390 and Arg391 are reported as the average ± standard deviation of variants for position 390 and 391, respectively. Potential substitutions of residues 390 and 391 due to point mutations are shown in italics.

Interestingly, such destabilizing effect of all variants was found also when considering αβ tubulin heterodimers, although in a variant-dependent manner. Indeed, the destabilization of the dimer due to the Tyr310His substitution was comparable to that of the isolated protein (∆∆G_f_^app^ = 3 vs 3.13 kcal/mol). On the other hand, disease-associated variants Arg390Gln and Arg390Trp, and Arg391Cys and Arg391His displayed an almost twofold larger destabilization of the dimeric complex with respect to that of the isolated protein (∆∆G_f_^app^ = 17.52, 19.08, 16.27, and 21.25 kcal/mol vs 8.27, 9.59, 10.81, and 9.26 kcal/mol, respectively, Table 2).

As to the affinity of TUBB4B for α-tubulin, the Tyr310His substitution was predicted not to affect dimer formation (∆∆G_b_^app^ = 0.01 kcal/mol, Table 2). On the contrary, amino acid substitutions at Arg390 and Arg391 were predicted to significantly decrease complex affinity (∆∆G_b_^app^>8.36 kcal/mol for all tested variants, Table 2) mainly due to altered Coulombic and hydrophobic interactions involving α-tubulin residues Tyr262, Trp346 and Ser349, and β-tubulin residue Phe394 (Fig. 8). Noteworthy, the Arg391Cys and Arg391His variants exhibited >twofold larger reduction in affinity with respect to the Arg390Gln and Arg390Trp variants, suggesting a more detrimental molecular phenotype.

Similar conclusions could be drawn when considering all potential substitutions of residues Arg390 and Arg391 (Arg390 and Arg391 in Table 2). Indeed, no statistically significant differences among the two positions could be identified in terms of destabilization of both the isolated protein (∆∆G_f_^app^ = 15.58 ± 17.83 vs 14.16 ± 9.57 kcal/mol, respectively, Table 2) and the heterodimeric complex (∆∆G_f_^app^ = 26.32 ± 18.23 vs 22.53 ± 7.63 kcal/mol, respectively, Table 2), in line with what was observed on each variant identified in this patient cohort. Despite the similar effect exerted by missense variants at amino acid position 390 and 391 on the protein/complex stability, a substitution of Arg391 was predicted to result in a more than twofold decrease in heterodimer affinity compared to a substitution of Arg390 (∆∆G_b_^app^ = 21.04 ± 2.87 vs 10.25 ± 1.73 kcal/mol, respectively, Table 2), again in line with the behavior exhibited by each identified variant.

Overall, our results show that the missense variants found in this patient cohort are associated with a peculiar clinical phenotype that depends on the position of the mutated residue, although a general destabilization of either the individual TUBB4B protein or its interaction with α-tubulin was observed for each variant.

## Discussion

In our study, we report on six patients with likely *TUBB4B*-associated IRD and SNHL, or LCAEOD.

### Ophthalmologic phenotype

Pathogenic variants in *TUBB4B* have only recently been associated with an autosomal dominantly inherited form of early retinal degeneration and hearing loss^3^. Only amino acid substitutions at codon 391 (c.1171C>T;p.(Arg391Cys) and c.1172G>A;p.(Arg391His)) have been described^3–5^. In our cohort, both known variants were carried by one patient each. Luscan et al. reported five patients from four families with retinal pathology including features such as yellowish peripheral retina with round pigmented spots, thin retinal vessels, and macular alterations^3^. Medina et al. reported two patients with early onset vision and hearing loss without a detailed description of the retinal phenotype^4^. Maasz et al. reported three patients from a single family with a phenotype similar to that reported by Luscan et al.^3,5^. Patient 5 in our cohort had corresponding fundus findings. All the patients mentioned here had in common that the changes occurred panretinally and not in a sectorial pattern. Secondly, none of these previously reported patients exhibited bone spicules. Patient 4 of our cohort was special, as he genetically presented with mosaicism (24% allelic reads). Ophthalmologically, he showed a less symmetrical but sectorial retinal disease pattern with both areas of physiologically appearing retina adjacent to areas of pathological retinal tissue with bone spicules in the more peripheral lesions—likely due to the mosaicism which was further strongly supported by the results of the extended molecular genetic analysis (Supplementary Fig. S1). Luscan et al.^3^ presented a similar case in which the mother of one of the patients with *TUBB4B*-associated LCA and SNHL carried 29% of mutant reads^3^. In this subject, disease was milder than in the other patients (i.e. less symptomatic and with a later onset) and fundus examination also showed a sectorial pattern and bone spicules. This patient can well be compared to Patient 4 in our cohort. From all this, we firstly conclude that patients with amino acid substitutions at position 391 of the TUBB4B protein exhibit a distinct retinal phenotype with round yellowish peripheral lesions with pigmented spots, corresponding to LCA and SNHL, and secondly, that patients with mosaicism at this position show a sectorial pattern with the presence of bone spicules and a milder disease course.

The three patients in our cohort with an amino acid substitution at position 390 of the TUBB4B protein consistently exhibited a completely different retinal phenotype: Patient 1 and Patient 2 are son and mother, and both carried the variant p.(Arg390Gln), exchanging the arginine with glutamine; Patient 3 is a sporadic case presenting with the exchange of arginine with tryptophan p.(Arg390Trp). Pathogenic variants in the respective codon have not been reported so far. All three patients showed a pattern of so-called pericentral RP in which the tissue affected is located rather centrally (extending from the parafovea to the arcades) in comparison to typical RP, which naturally displays the most early or extensive changes in the midperiphery^10–12^. Additionally, the atrophic lesions showed up as well-demarcated with significantly decreased autofluorescence, adjacent to areas with physiologic autofluorescence on FAF imaging in all three patients^13^. Patient 1 and Patient 3 had preserved foveal tissue allowing for good visual acuity. Patient 2 had retained good visual acuity until her late 40s keeping in line with the phenotype of Patient 1 and Patient 3. Here, we conclude that patients with amino acid substitutions at position 390 of the TUBB4B protein exhibit a second distinct retinal phenotype, namely a well-demarcated pericentral RP, which, to our knowledge, we describe for the first time.

As patients with missense variants at Arg390 exhibited a clearly different retinal phenotype from those harboring missense variants at Arg391, we investigated whether such differences may correspond to a different molecular phenotype. All disease-associated variants identified in this patient cohort were found to destabilize the folding of isolated TUBB4B and to decrease both the affinity and the structural stability of the αβ tubulin heterodimer, though to a different extent. Despite Arg390 and Arg391 substitutions showing a similar destabilization of both isolated and complexed TUBB4B, the decrease of the apparent affinity of the αβ tubulin heterodimer associated with such disease-associated variants was significantly different. Indeed, the novel Arg390Gln and Arg390Trp variants identified in this study exhibited a halved reduction in apparent affinity compared to that shown by patients harboring the previously identified Arg391Cys and Arg391His variants^3^, suggesting an association with the milder retinal phenotype. Moreover, since αβ tubulin heterodimers constitute the repeating unit within microtubules (Fig. 1B), such effects on heterodimer affinity and stability indicate that the stability of the microtubule may be compromised by the propagation of the destabilization of periodic αβ tubulin assembly.

The effects of the Tyr310His variant on the stability of both isolated and complexed TUBB4B, on the other hand, were more than threefold smaller compared to the substitutions of either Arg390 or Arg391, with no repercussions on αβ tubulin affinity. Thus, since Tyr310 is known to possibly be phosphorylated^14^, our results suggest that the pathological mechanism for the Tyr310His variant, responsible for a peculiar clinical phenotype, may involve an altered phosphorylation pattern. Furthermore, this hypothesis might also explain the very early-onset phenotype exhibited by Patient 6, as no information about the functional role of phosphorylated Tyr310 in TUBB4B is available so far and will be investigated in potential functional studies. While the auditory phenotype of this young patient could well be established, ophthalmological examination was limited. Reduced responses in ERG testing provided a hint towards global retinal pathology, yet follow-up examinations are warranted to establish a more detailed ophthalmological diagnosis. Additionally, reclassification of the respective variant should be attempted on a medium term, which remains as of now a variant of uncertain significance and association of this variant to the patient’s phenotype can neither be proven, nor be discharged.

All cases of patients with a *TUBB4B* disease-associated variant reported so far showed hyperopia^9^. Also four of our six patients showed hyperopia. Explanatory approaches see the importance of microtubules for a correct cell migration in the lens tissue causal for this^9^.

### Auditory phenotype

The auditory phenotype of the patients in our cohort showed a wide range of hearing loss grades and frequency spectra. Irrespective of higher loss grades and frequency distribution, all patients demonstrated bilateral symmetric hearing loss, typical for most other forms of genetic hearing loss^15,16^. The hearing loss grades were distributed from a near-normal hearing loss grade to a complete hearing loss grade. The frequency spectra included pan-cochlear and downsloping high-frequency configurations. As for genotype–phenotype correlations, the two related patients (1 and 2, son and mother) with the variant p.(Arg390Gln) showed different grades of hearing loss. Additionally, the patient (4) with the variant p.(Arg391His) demonstrated a mild hearing loss grade while the same genotype has been described for five patients in another cohort^3^ exhibiting variable hearing loss grades including mild, moderate, severe and complete hearing loss. This confirms that the grade of hearing loss is highly variable in *TUBB4B*-related patients even for the same variant. Therefore, hearing loss grades in *TUBB4B*-related patients may depend on other endogenic or exogenic factors.

### Extended genetic analyses

Genetic reinvestigation of seven additional unsolved USH patients from our database excluded variants at codons Arg390/Arg391 in these subjects, hereby underpinning that variants in *TUBB4B* are an ultra-rare cause of combined retinal disease and hearing loss.

## Material and methods

### Study design

In this study, we collected data on the ocular and audiological phenotype and genotype of patients with *TUBB4B*-associated IRD and SNHL. The study was conducted in accordance with the Declaration of Helsinki, with approval from the ethics committee of the Medical Faculty of the University of Tübingen (study no. 116/2015BO2). Informed consent war obtained from all participants or their legal guardians. All examinations were performed at the University Clinics of Tübingen, Germany, specifically the Centre for Ophthalmology, the Department of Otolaryngology-Head & Neck Surgery and the Institute of Medical Genetics and Applied Genomics, all tertiary referral centers for rare diseases.

### Patients

All patients heterozygous for a (likely) disease-causing variant in *TUBB4B* who presented to our Inherited Retinal Disease Consultation or to the Department of Otolaryngology-Head & Neck Surgery since January 2019 were included in the study.

### Ophthalmological assessments

Ophthalmic examination included best corrected visual acuity (BCVA), slit lamp and dilated fundus examination, fundus photography, spectral domain optical coherence tomography (OCT) and fundus autofluorescence (FAF) imaging (Spectralis^®^ HRA + OCT, Heidelberg Engineering GmbH, Heidelberg, Germany), semi-automated 90° kinetic visual field (VF) exam using target III4e (Octopus 900, Haag-Streit, Wedel, Germany), and full field electroretinogram (ffERG) testing according to the International Society for Clinical Electrophysiology of Vision (ISCEV) standards (Espion, Diagnosys, Lowell, MA, USA). In patient 06, ffERG was performed under general anesthesia (total intravenous anaesthesia, Remifentanil, Propofol) using the RETeval device and skin electrodes (LKC Technologies, Gaithersburg, MD, USA). BCVA was converted to Snellen ratio^17^.

### Audiological assessments

The audiological assessment was performed with a standard audiometer (AT900 or AT1000; AURITEC Medizindiagnostische Systeme GmbH, Hamburg, Germany) equipped with air- and bone conduction headphones (DT48; Beyerdynamic, Heilbronn, Germany).

The pure tone average threshold for frequencies of 0.5, 1, 2, and 4 kHz (PTA4) was assessed for each ear in air and bone conduction. Grades of hearing impairment were classified as PTA4 in seven mutually exclusive severity categories as recommended by the GBD Expert Group on Hearing Loss^18,19^. Accordingly, PTA4 results for single ears of all probands were classified into hearing loss grades designated as normal hearing (− 10.0 to 19.9 dB HL), mild hearing loss (20.0 to 34.9 dB HL), moderate hearing loss (35.0 to 49.9 dB HL), moderately severe hearing loss (50.0 to 64.9 dB HL), severe hearing loss (65.0 to 79.9 dB HL), profound hearing loss (80.0 to 94.9 dB HL) and complete or total hearing loss (≥ 95.0 dB HL).

Speech discrimination was assessed with the Freiburg monosyllabic speech test^20^ for the German language at 65 dB and 80 dB sound pressure level (SPL) in quiet to determine the word recognition score (WRS) at 65 dB (WRS65) and at 80 dB (WRS80), respectively, including contralateral masking if applicable, such as in single-sided deafness or contralateral residual hearing^21,22^. The Freiburg speech test is the standard reference speech test in quiet for the German language in German-speaking countries and has been widely used over more than five decades^20,21,23–26^. The Freiburg monosyllable speech test consists of 20 phonemically balanced monosyllable word lists with 20 items each. Normal-hearing subjects achieve a WRS of 50% at a presentation level of 30 dB SPL (WRS30) and 100% at 50 dB SPL (WRS50)^27^. For hearing-impaired subjects, typically, the WRS65 is evaluated. In one patient speech recognition was assessed with the Göttingen speech perception test which is an open set monosyllabic word test designed for children. The children have to repeat 10 monosyllable words (10% per correct word) presented by loudspeaker^28^. The infant underwent brainstem audiometry using Path medical's Sentiero advanced with a chirp stimulus and levels in the air conduction up to 90 dB and in the bone conduction up to 60 dB.

### Genetic testing

Genetic diagnostic testing of patients in this study was performed by disease gene panel analysis (patient 1, 3, 4 and 5: 565, 577, 558 and 576 syndromic and non-syndromic IRD genes, respectively; patient 6 222 SNHL genes) based on genome sequencing^29^, except for patient 2, mother of patient 1, who was solely genotyped for the *TUBB4B* c.1169G>A;p.Arg390Gln variant by PCR amplification of exon 4 out of genomic DNA and Sanger sequencing in a research context. Genomic coordinates given in this manuscript are based on the GRCh38 genome (hg38). Variant nomenclature is in accordance with Human Genome Variation Society recommendations^30^ and based on GenBank accession numbers NM_006088.6 and NP_006079.1 and Ensembl reference ENST00000340384.5 with nucleotide one being the first nucleotide of the translation initiation codon ATG. In addition to this diagnostic genetic work-up via genome sequencing, genomic DNA of seven patients with a clinical diagnosis of Usher syndrome but unresolved genetic cause were analyzed by PCR amplification of exon 4 and Sanger-sequencing, but none of these cases carried a variant at codon 390/391. Also segregation analysis in both parents of patient 4 was done by PCR amplification of exon 4 and Sanger-sequencing.

### Molecular modeling of human TUBB4B and structural analysis of pathological variants

The three-dimensional structure of human tubulin beta-4B chain was obtained by homology modeling, using as template the crystallographic structure of bovine tubulin beta-2B chain (sequence identity 96.9%) with Protein Data Bank (PDB) identifier 4I4T^31^ (resolution 1.8 Å), by selecting the beta-chain with the highest percentage of solved residues (chain D in the PDB file), and retaining the respective ligands (Mg^2+^, Ca^2+^, and GDP). Homology modeling, structure refinement procedures, and calculation of the differences in Gibbs free energy due to in silico mutagenesis were carried out using the BioLuminate interface provided by Maestro molecular modeling suite (Schroedinger, New York, USA).

Briefly, the structure of isolated TUBB4B was subjected to the protein preparation wizard provided by BioLuminate, which assigned bond orders based on the Chemical Components Dictionary database (http://www.pdb.org, wwPDB foundation, Piscataway NJ, USA), added H-atoms, created zero-order bonds to metals, and generated protonation states for GDP and protein at pH 7.5 using Epik and PROPKA^32^ modules, respectively. After assigning and optimizing the geometry and distances for H-bonds, structures were minimized using Optimized Potentials for Liquid Simulations 4 (OPLS4) forcefield^33^, setting 0.3 Å as the heavy-atoms Root-Mean Square Deviation (RMSD) threshold for reaching convergence.

The αβαβ organization of tubulin chains was reconstituted using the structure with PDB identifier 4I4T^31^ as template, which contains the coordinates of the tubulin (αβαβ chains) - stathmin-4 - tubulin tyrosine ligase complex, upon superimposition of the model of the isolated TUBB4B on the two tubulin beta-2B subunits (chains B and D in the PDB entry), with a Cα-RMSD of 0.395 and 0.001, respectively.

The tubulin αβ and βα heterodimers were extrapolated from the complex to evaluate in detail the interactions of TUBB4B with the preceding or following α subunits.

All potential substitutions due to point mutations in the codons encoding for residues Arg390 (to Gln, Gly, Leu, Pro, and Trp) and Arg391 (to Cys, Gly, His, Leu, Pro, and Ser), as well as the Tyr310His mutation were introduced by the BioLuminate’s Residue scanning tool for in silico mutagenesis. The most naturally occurring rotamer was selected for each side chain substitution, then all structures underwent energy minimization with the same computational parameters as above.

The thermodynamic cycle for each TUBB4B variant was computed according to the Molecular Mechanics/Generalized Born and Surface Area Continuum solvation (MM/GBSA) method^34^. This method, which allows the evaluation of the differences in Gibbs free energy of folding (∆∆G_f_^app^ = ∆G_f_^app^_mut_ − ∆G_f_^app^_WT_) and binding to Tubulin α-1B chain (∆∆G_b_^app^ = ∆G_b_^app^_mut_ − ∆G_b_^app^_WT_) with respect to the wildtype does not consider the explicit energetic term associated with the conformational change. Therefore, the differences in free energy reported in this study cannot be considered precise thermodynamic values, but they represent variations in apparent stability (∆∆G_f_^app^) or affinity (∆∆G_b_^app^), which nevertheless have been proven to correlate with functional data also in other protein systems^35^.

### Institutional review board statement

The study was conducted according to the guidelines of the Declaration of Helsinki and approved by the Ethics Committee of the University Hospital of Tübingen under the study number 116/2015BO2.

### Informed consent statement

Informed consent was obtained from all subjects involved in the study or their legal guardians. Written informed consent was obtained from the patients or their legal guardians to publish this paper.

## Conclusions

Pathogenic variants in *TUBB4B* have been associated with an autosomal dominantly inherited form of early retinal degeneration and hearing loss. Patients with amino acid substitution at position 391 of the TUBB4B protein show round yellowish peripheral lesions with pigmented spots, corresponding to LCA and SNHL. Patients with amino acid substitutions at position 390 of the TUBB4B protein reveal a well-demarcated pericentral RP. Variants at codon positions 390 and 391 were predicted to decrease the structural stability of TUBB4B and its complex with α-tubulin, as well as the complex affinity. The twofold larger reduction in heterodimer affinity exhibited by Arg391 substitutions suggested an association with the more severe retinal phenotype, compared to the substitution at Arg390. The effects of the Tyr310His variant on the stability of both isolated and complexed TUBB4B were more than threefold smaller compared to the substitutions of either Arg390 or Arg391, with no repercussions on αβ tubulin affinity. This suggests that the Tyr310His variant might be associated with a different phenotype through an undisclosed mechanism. The grade of hearing loss is highly variable in *TUBB4B* patients even for the same variant. Therefore, hearing loss grades in *TUBB4B* patients may depend on other endogenic or exogenic factors.

### Supplementary Information

Supplementary Figure S1.

## Data Availability

The data presented in this study is contained within the article and supplementary material. The cases were submitted to ClinVar and the following submission IDs were assigned: SUB14379232, SUB14379238, SUB14379294, and SUB14379295. In one case, the accession number is already available: SCV001905645.1.
