# Supplementary material for: Comprehensive analysis of two hotspot codons in the TUBB4B gene and associated phenotypes
Source: Sci Rep. 2024 May 8;14:10551. doi: 10.1038/s41598-024-61019-0 (PMC11078972; doi:10.1038/s41598-024-61019-0)
Supplement: Supplementary file 1 — Supplementary Figure S1. [file 41598_2024_61019_MOESM1_ESM.pdf]

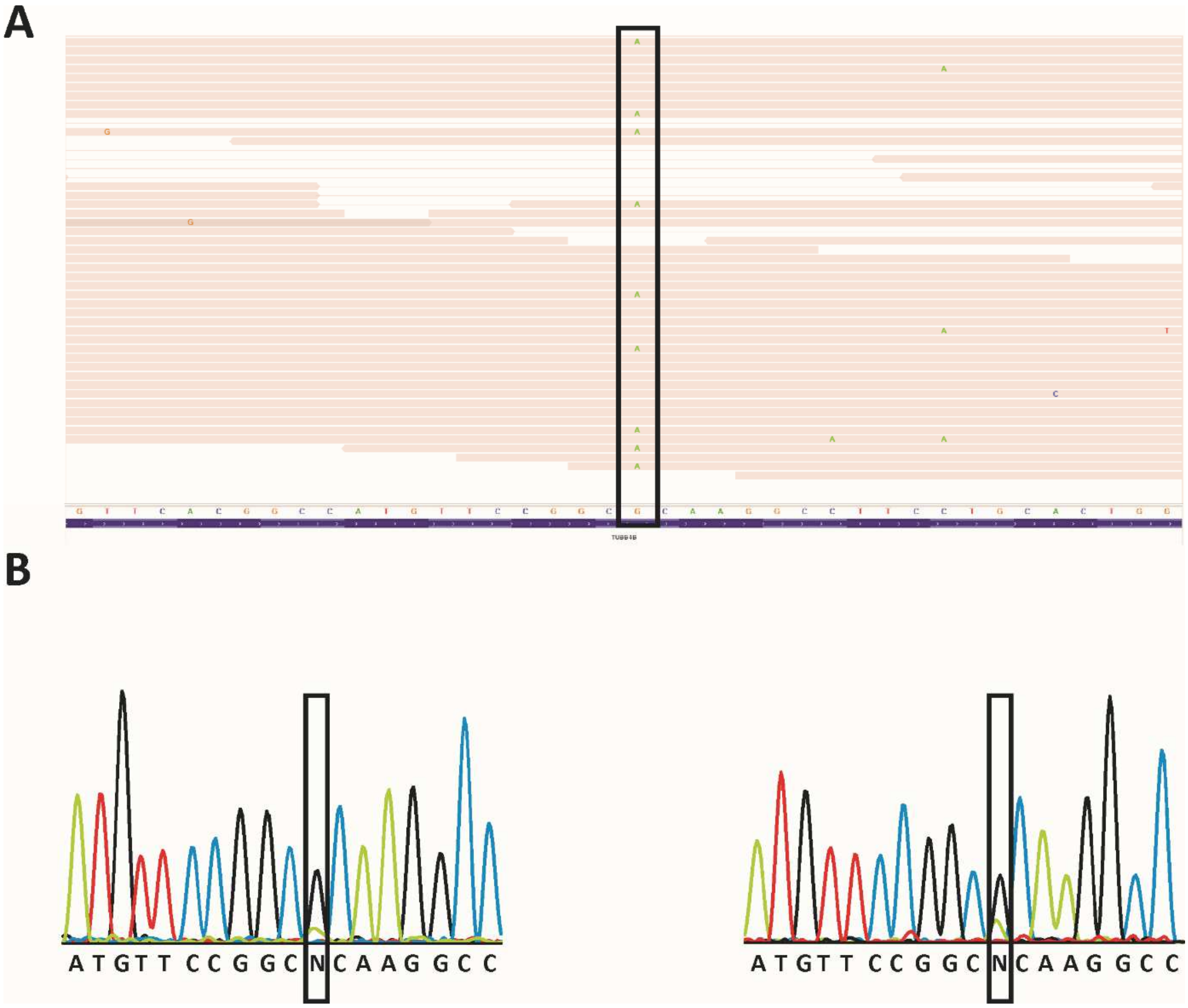

Supplementary Figure S1. Genomic testing of patient 4. (A) Genome sequencing reads visualized in the Integrative Genomics Viewer (IGV), showing the TUBB4B variant c.1172G>A;p.(Arg391His) in a mosaic state with a calculated variant allele frequency of ~24%. (B) Sanger sequencing confirmation of the mosaic variant. Sequence electropherograms were obtained with a forward (left) and a reverse primer (right).
